# Follow-Up After Urgent Endodontic Care: A Retrospective Exploratory Study in a University Clinic

**DOI:** 10.3390/medicina62030544

**Published:** 2026-03-14

**Authors:** Mubashir Baig Mirza, Turky AlShammeri, Saleh AlMutairi, Shahad AlBader, Abdulaziz Abdulwahed, Osamah AlQasem, Ahmed AlMokhatieb, Laila S. AlMufleh, Mohammed Ali Abuelqomsan, Qamar Hashem

**Affiliations:** 1 Conservative Dental Sciences, College of Dentistry, Prince Sattam bin Abdulaziz University, Al-Kharj 11942, Saudi Arabia; s.albader@psau.edu.sa (S.A.); a.abdulwahed@psau.edu.sa (A.A.); o.alqasem@psau.edu.sa (O.A.); a.almokhatieb@psau.edu.sa (A.A.); l.almufleh@psau.edu.sa (L.S.A.); m.abuelqomsan@psau.edu.sa (M.A.A.); q.hashem@psau.edu.sa (Q.H.); 2 College of Dentistry, Prince Sattam bin Abdulaziz University, Al-Kharj 11942, Saudi Arabia; 442051303@std.psau.edu.sa (T.A.); 442051446@std.psau.edu.sa (S.A.)

**Keywords:** endodontics, patient compliance, root canal therapy, urgent care

## Abstract

*Background and Objectives:* Many patients present with urgent endodontic conditions characterized by pain and swelling, requiring prompt intervention. Failure to return for definitive root canal treatment (RCT) after urgent care can compromise tooth prognosis. This study examined the frequency and diagnostic patterns of urgent endodontic visits at a university dental college and evaluated predictors of follow-up after urgent treatment, considering demographic, anatomical, and clinical factors. *Materials and Methods:* A retrospective cohort analysis of 1619 patient records (August 2023–May 2025) was conducted. Data on urgency type, pulpal/periapical diagnosis, tooth characteristics, urgent treatment modality, and follow-up attendance were collected. Descriptive statistics and chi-square tests were used to assess bivariate associations. Multivariable logistic regression was performed to evaluate factors associated with completing follow-up after urgent care. *Results:* Approximately 50.2% of visits involved urgent endodontic care, most commonly symptomatic irreversible pulpitis (SIP) with symptomatic apical periodontitis (SAP), particularly in posterior teeth. Pulpectomy was the most frequently provided urgent treatment. Overall, 72.8% of urgent-care patients returned for completion of RCT (overall OR = 2.67). While urgency incidence and follow-up rates did not differ by gender, arch, or region, gender-stratified comparisons within urgent cases showed significant differences by arch and region. In multivariate analysis, mandibular teeth demonstrated higher odds of follow-up than maxillary teeth, whereas gender and region showed no association. Patients diagnosed with asymptomatic irreversible pulpitis (AIP)/SAP had lower odds of returning, and those treated with canal debridement with occlusal reduction (CD/OD) were also less likely to complete treatment. No other diagnostic or treatment categories showed significant associations. *Conclusions:* In this university-based cohort, urgent endodontic visits were common and primarily involved SIP/SAP. While the overall follow-up rate was high, certain diagnostic and treatment scenarios, specifically AIP/SAP and CD/OD, were associated with a reduced likelihood of returning for completion of RCT. Given the study’s limitations, these findings should be interpreted with caution.

## 1. Introduction

The root canal and periapical tissues are the primary sites of infection and inflammatory disorders of the teeth. These often painful and infectious conditions present significant challenges for both patients and dental care providers [1]. Endodontic problems account for 60–82% of dental emergencies, with symptomatic irreversible pulpitis (SIP) being the most common reason for urgent care. Approximately 60% of SIP cases also present with symptomatic apical periodontitis (SAP) [2]. SIP typically causes dull, throbbing, persistent pain that may occur spontaneously or be triggered by heat, cold, or chewing, whereas SAP produces marked tenderness on biting, percussion, or palpation [3]. An acute apical abscess (AAA) represents rapid progression of the same disease process and is characterized by severe pain, swelling, pus formation, extreme tenderness, sometimes accompanied by fever, malaise, and lymphadenopathy [4].

In clinical practice, emergencies require immediate assessment and treatment during unscheduled visits, whereas urgent conditions are less severe and can be scheduled more flexibly [5]. However, the American Association of Endodontics (AAE) classification system does not distinguish symptomatic cases by severity, as the diagnostic approach and treatment needs remain consistent regardless of whether the presentation is urgent or emergent [1]. SIP, SAP, and AAA represent distinct biological phases of continuously evolving bacterial infections, and without timely intervention, may spread to surrounding tissues and, in severe cases, lead to life-threatening complications [1,6]. These conditions frequently disrupt daily activities, cause patients to miss work, and necessitate operative intervention for pain relief [7,8]. The primary purpose of urgent intervention is to rapidly and effectively reduce pain until definitive treatment can be completed, while preventing persistent pain, periapical pathology, and systemic complications [3].

Although complete instrumentation and placement of an intracanal dressing are recommended, limited time during unscheduled visits often necessitates initial therapeutic interventions [9,10]. Historically, pulpectomies (Pp) were preferred; however, recent studies show that coronal pulpotomies (Po) with calcium silicate capping materials can similarly alleviate pain in SIP [11]. Occlusal reduction also significantly reduces pain in patients with vital pulps, periradicular symptoms, and pre-operative discomfort. In cases of abscess, incision and drainage (I&D) reduces microbial load and inflammation, thereby preventing the spread of infection [7]. Patients often seek urgent care for pain but may not return for further treatment once the symptoms subside. Failure to complete treatment poses significant risks, as urgent interventions without subsequent care can lead to recurrent symptoms and complications [12]. Delaying permanent restorations increases the likelihood of coronal leakage, treatment failure, and tooth fracture, which may complicate future procedures and potentially render the tooth non-restorable [13]. At a US military school, 56% of incomplete root canal treatments (RCTs) eventually required extraction [14]. Choosing extraction over RCT has also been shown to negatively affect patients’ quality of life [15].

Previous research has primarily focused on urgent endodontic treatments aimed at relieving pain, with limited attention to patterns of urgent presentations and procedures performed by dental students in a university setting. A recent study of dental interns highlights notable variation in their emergency treatment choices, reflecting inconsistent understanding of urgent care protocols [16]. Although urgent care can provide rapid symptom relief and may reduce patients’ perceived need for follow-up visits, the factors that deter patients from returning remain poorly understood [17]. Only a few studies have examined RCT completion rates after urgent care, typically evaluating individual predictors such as age, gender, insurance, number of visits, and tooth type [17,18]. To date, no study has assessed how demographic characteristics, anatomical factors, diagnostic categories, and urgent treatment modalities jointly influence patient compliance with treatment completion within a multivariable framework. This study addresses this gap by evaluating follow-up among patients receiving urgent endodontic treatment in a university-based setting and examining whether patient-level factors (gender), anatomical factors (arch and region), diagnostic category, and urgent intervention type are associated with completion of RCT.

## 2. Materials and Methods

### 2.1. Study Design and Sampling

This study employed a retrospective cohort design and used convenience sampling from patient records within the institutional database, following defined inclusion and exclusion criteria. Because care at the university clinic is provided free of charge, patient attendance may be higher, introducing a structural confounder that could influence follow-up rates. Therefore, the findings should be considered setting-specific and exploratory.

### 2.2. Ethical Statement

The study was conducted in accordance with the Helsinki Declaration. Ethical approval was obtained from the Bioethics Research Board at Prince Sattam bin Abdulaziz University (approval number SCBR-420/2025, dated 9 February 2025).

### 2.3. Data Collection and Sample Characteristics

Patient data were collected from 20 August 2023 to 30 May 2025, using the EXACT Dental software (Version 2020, Henry Schein One, Auckland, New Zealand). Clinical records and treatment notes were reviewed to identify all patients who underwent RCT during this period. Before data collection, all reviewers were trained on the study protocol, terminology, and data entry procedures. Two researchers independently reviewed each record, categorizing urgent RCT cases by pulpal and periapical diagnosis and by the type of therapeutic treatment provided. A binary response (yes/no) was recorded for urgency, along with the tooth number and patient gender. A similar binary response was documented for follow-up attendance on the same tooth within the scheduled appointment date. Two additional researchers verified the extracted data using the same criteria, organizing records by file number to ensure accuracy and reliability. Only files with mutually agreed-upon conclusions were included, and any disagreements were resolved through consensus. Inter-reviewer reliability was assessed using Cohen’s kappa coefficient, which demonstrated excellent agreement (κ = 0.824).

### 2.4. Diagnostic and Treatment Patterns

All patients requiring urgent endodontic care were diagnosed according to the AAE classification summarized in Table 1. Therapeutic management followed diagnostic patterns and included Pp, Po, canal debridement (CD) (with or without occlusal reduction) (OD), retreatment (ReTx) (with or without OD), and CD with I&D. Table 2 provides brief explanations of these procedures. Final data were anonymized and categorized by gender, region, arch, diagnosis, and treatment modality. All variables were coded and prepared for statistical analysis.

### 2.5. Sample Size Calculation

A priori sample size estimation was performed using G*Power version 3.1.9.2 for a binary logistic regression model with follow-up status as the dependent variable. Assuming a medium effect size (odds ratio of 1.5), a significance level of 0.05, and a power of 0.80, the required minimum sample size was 128 participants. The final sample included 812 urgent cases, substantially exceeding the requirement and ensuring adequate power to detect clinically meaningful associations.

### 2.6. Inclusion and Exclusion Criteria

This study examined patients who underwent urgent RCT at the College of Dentistry between 20 August 2023 and 30 May 2025. Inclusion criteria included patients presenting for urgent endodontic care on permanent teeth, for whom a follow-up appointment was indicated to complete RCT, and who had complete documentation available for gender, tooth number, diagnosis, treatment rendered, and follow-up status. Exclusion criteria included urgent cases involving dental trauma, treatments performed on primary teeth, and cases in which definitive RCT was completed in a single visit. Additionally, records with incomplete documentation, patients referred externally immediately after urgent care, and cases managed solely with pharmacologic therapy without operative intervention were excluded. Patients who returned for their scheduled appointments (based on clinic availability) to complete treatment were classified as follow-up cases, whereas those who did not attend their recommended visit were categorized as non-follow-up patients.

### 2.7. Statistical Analysis

Data were entered and analyzed using SPSS version 26 (SPSS Inc., Chicago, Ill., USA). Descriptive statistics (frequencies and percentages) were generated for all categorical variables, including gender, arch, region, diagnostic categories, and treatment modalities. Bivariate associations were assessed using the chi-square test, with *p* ≤ 0.05 considered statistically significant. Multivariable logistic regression analyses were conducted to identify independent predictors of follow-up completion, and adjusted odds ratios (aORs) with 95% confidence intervals (CIs) were reported. For the diagnosis-based model, penalized (ridge) logistic regression was used because several low-frequency diagnostic categories induced quasi-separation and prevented convergence under standard maximum-likelihood estimation. Bootstrap percentile CIs (B = 200) were generated for the penalized estimates, and model calibration was assessed using the Hosmer–Lemeshow goodness-of-fit test. For the treatment-based multivariable analysis, a non-penalized logistic regression model was fitted after removing the I&D group, as it caused anatomic sparsity and hindered model convergence. Model performance was evaluated with Nagelkerke R^2^ and the Hosmer–Lemeshow test. Additionally, unadjusted logistic regression models were used to estimate overall odds ratios for follow-up, stratified by diagnostic category and urgent treatment modality. These models used follow-up status as the outcome and diagnosis and treatment category as predictors, with 95% CIs calculated through standard maximum-likelihood estimation.

## 3. Results

**Figure 1:**
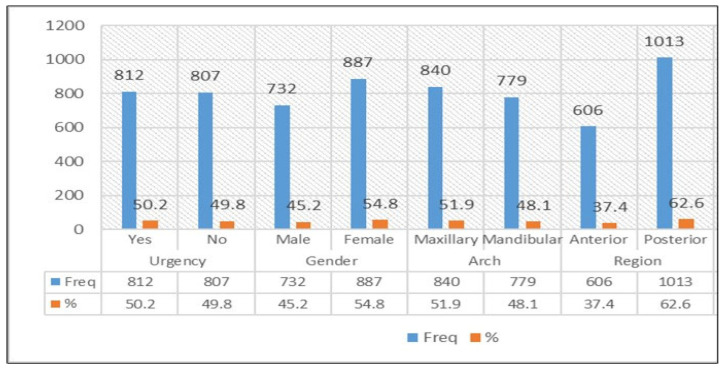
Distribution of cases by urgency status, gender, arch, and region.

A total of 1619 patients received endodontic treatment during the study period. Of these, 50.2% (n = 812) were classified as urgent. The distribution of cases by urgency status, gender (male/female), arch (maxilla/mandible), and region [anterior (incisors and canines)/posterior (premolars and molars)] is shown in Figure 1.

Among urgent cases, 53.4% were female and 46.6% were male. Maxillary teeth accounted for 52.8%, and 62.9% involved posterior teeth, as shown in Table 3. A chi-square test showed no significant differences in urgency status across gender, arch, and region (*p* > 0.05).

When gender differences were examined within anatomical categories (Table 4), a statistically significant association was observed for arch (*p* = 0.031). Females presented more frequently with mandibular urgencies, whereas males more often presented with maxillary urgencies. Region also showed a significant association with gender (*p* < 0.001): females more frequently had posterior urgent cases, whereas males more often presented with anterior ones.

**Figure 2:**
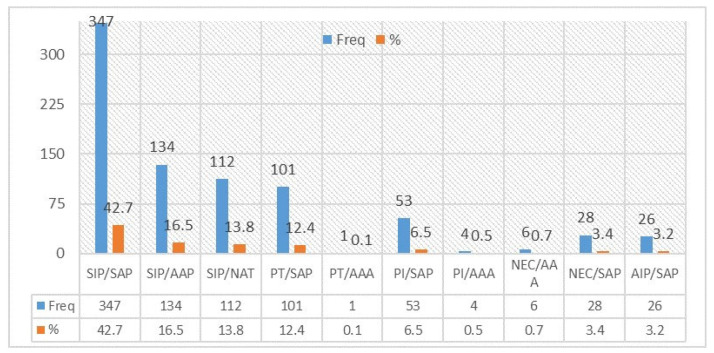
Diagnostic patterns of urgent endodontic cases.

Diagnostic patterns for urgent presentations are summarized in Figure 2. SIP was the predominant pulpal diagnosis (73%), most commonly associated with SAP (42.7%). SIP/AAP and SIP/NAT accounted for 16.5% and 13.8%, respectively. Previously treated (PT) cases represented 12.5%, previously initiated (PI) 7%, and necrotic pulp (NEC) 4.1% of urgencies. AIP/SAP comprised 3.2%.

Table 5 shows that diagnostic distribution differed significantly by region (*p* = 0.006), but not by gender or arch.

**Figure 3:**
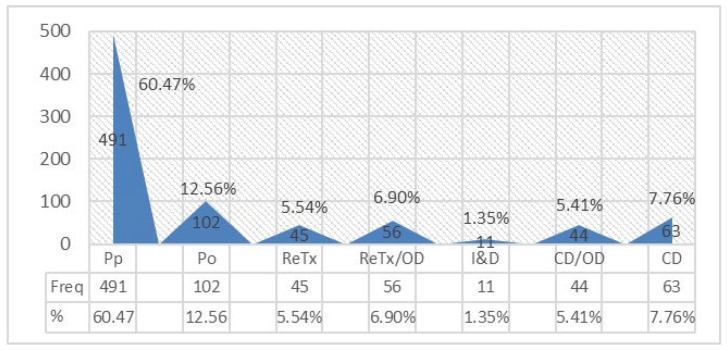
Treatment patterns for urgent cases.

Treatment patterns for urgent care are shown in Figure 3. Pp was the most common treatment (60.47%), followed by Po (12.56%), ReTx (5.54%), and ReTx/OD (6.90%). CD accounted for 7.76%, CD/OD for 5.41%, and I&D for 1.35%.

Table 6 shows no significant gender-based variation (*p* = 0.866). In contrast, arch (*p* < 0.001) and region (*p* < 0.001) were significantly associated with treatment modality. Maxillary teeth more often received Pp, ReTx, and CD, whereas mandibular teeth more often received Po and ReTx/OD. Anterior teeth were more frequently treated with ReTx and I&D, while posterior teeth more often received Pp, Po, ReTx/OD, and CD/OD.

Table 7 presents multivariable findings from the ridge-penalized logistic regression model. Mandibular teeth had higher odds of follow-up (aOR = 1.57; 95% CI: 1.16–2.07). Gender and region were not significant predictors. Compared with SIP/SAP, AIP/SAP was associated with lower odds of follow-up (aOR = 0.61; 95% CI: 0.24–1.00). The model demonstrated acceptable calibration (Hosmer–Lemeshow *p* = 0.697).

Table 8 presents results for the non-penalized model, which included treatment modality. Mandibular teeth again showed significantly higher odds of follow-up (aOR = 1.68; 95% CI: 1.21–2.34). Treatment type was significantly associated: CD/OD was linked to substantially lower odds of follow-up compared with Pp (aOR = 0.29; 95% CI: 0.15–0.55; *p* < 0.001). Other treatments did not differ significantly from Pp. The model showed good calibration (Hosmer–Lemeshow *p* = 0.724) and modest explanatory power (Nagelkerke R^2^ = 0.048). I&D was excluded from the non-penalized model because zero or sparse anatomical strata (mandibular arch and posterior region) would have prevented model convergence.

Follow-up rates by diagnosis are summarized in Table 9. Overall, 72.8% of urgent patients returned for follow-up. Diagnoses associated with SIP (SIP/SAP and SIP/NAT) had the highest odds of follow-up (OR = 3). Overall, patients requiring urgent RCT were 2.67 times more likely to return for follow-up care. AIP/SAP had the lowest follow-up odds (OR = 0.44; 95% CI: 0.20–0.96; *p* = 0.032). NEC/SAP showed a lower likelihood of follow-up, but the difference did not reach statistical significance (*p* = 0.063). Categories with small sample sizes (PT/AAA, PI/AAA, and NEC/AAA) produced wide confidence intervals, limiting interpretation.

Table 10 shows follow-up odds by urgent treatment modality. Pp was associated with a higher likelihood of follow-up (*p* = 0.042). Po and ReTx/OD had follow-up rates similar to the overall average (*p* > 0.90). CD/OD again showed significantly lower odds of follow-up (*p* < 0.001), consistent with multivariable findings.

## 4. Discussion

Most patients seeking endodontic treatment presented with pain-related urgencies, predominantly SIP, frequently accompanied by SAP. Overall urgency rates did not differ significantly by gender, arch, or region. However, within urgent cases, gender-stratified comparisons revealed notable differences across both arch and region. Diagnostic distribution also varied by region, although no significant differences were observed by gender or arch. Regarding urgent treatments, Pp was the most common intervention, and treatment patterns differed by arch and region. Patterns of follow-up behavior exhibited several consistent trends. Overall, 72.8% of urgent patients returned, with an unadjusted odds ratio of approximately 2.67. Multivariate analysis demonstrated that mandibular teeth had higher adjusted odds of follow-up than maxillary teeth, whereas gender and region were not associated with follow-up. Compared with SIP/SAP, AIP/SAP showed lower adjusted odds of returning. When treatment type was included in the model, the mandibular association persisted, and CD/OD was associated with markedly lower adjusted odds of follow-up than Pp; other treatment categories did not differ significantly from Pp. Unadjusted analysis aligned with these patterns: AIP/SAP had the lowest follow-up odds among diagnostic groups, CD/OD showed significantly lower follow-up relative to the overall reference, whereas Pp was associated with slightly higher follow-up odds than overall. Collectively, these results suggest that demographic and most anatomical factors were not major determinants of return for care, whereas diagnosis (AIP/SAP) and treatment (CD/OD) may be relevant barriers, and arch (mandible) showed a consistent positive association with follow-up in adjusted models.

Painful and infectious endodontic urgencies remain challenging for both patients and clinicians. Pulpal pain is widely recognized as one of the most intense forms of dental discomfort and is a common reason for seeking emergency care, a pattern also evident in the present study. Consistent with previous reports showing that urgent endodontic visits are predominantly related to SIP and SAP, the majority of urgent cases in this study were diagnosed within these categories [17,19]. Posterior teeth, including both premolars and molars, accounted for most urgencies; this classification enabled clearer comparison between the anterior and posterior regions [2]. Although maxillary teeth were slightly more common than mandibular teeth in urgent presentations, the difference was not statistically significant, aligning with findings from Falcon et al., who also observed no arch-related difference in urgency incidence [17]. These findings emphasize that urgent endodontic cases are primarily driven by symptomatic pulpal and periapical pathologies rather than by demographic factors or anatomical variations.

Regarding gender-based differences, studies examining general dental emergencies have reported a slight male predilection [20]. However, research focused specifically on endodontic urgencies typically shows no significant gender differences in the likelihood of seeking urgent endodontic treatment, a pattern also observed in the present study [21]. Gender was not associated with the overall incidence of urgent visits or with the diagnostic distribution across pulpal or periapical categories. This contrasts with findings from an Australian study reporting higher rates of PI and AAP in females and greater PN cases among males [22]. Although gender did not influence the incidence or diagnosis of urgency in our cohort, further stratification by arch and region revealed notable differences in distribution. Females presented more frequently with mandibular and posterior urgent cases, whereas males more often presented with maxillary and anterior urgencies. While these differences are not clinically predictive, as they do not translate into differences in the incidence of urgency or clinical diagnosis, they may still offer insight into tooth-type susceptibility and patterns of seeking urgent care.

Complete cleaning and shaping to remove the inflamed pulp and reduce the bacterial load within the canals is considered the most appropriate treatment in such situations. However, due to patient-related factors and time constraints, Pp and Po are often performed. Both procedures have been shown to reduce postoperative pain by up to 90% [5,19]. In this study, Pp was the most frequently performed urgent treatment, and it was also associated with high follow-up rates. This differs from some reports, such as a study from Cork University in Ireland, which documented lower recall rates after urgent Pp [23]. Recent histological evidence suggests that the clinical diagnosis of irreversible pulpitis does not always reflect the true pulpal status, as inflammation may be confined to the coronal pulp, while radicular tissue retains healing potential. In light of advances in calcium silicate materials, these findings have renewed interest in Po [19,24]. Evidence indicates that Po provides pain relief comparable to Pp in SIP cases, with symptom reduction occurring within hours [25]. Long-term outcomes are also promising, with a recent randomized controlled trial reporting an overall success rate of 83.5%, with particularly favorable results for partial Po [26]. Despite this growing evidence, Po accounted for only 12.6% of urgent treatments in the present study, lower than the approximately 25% reported in other studies [27]. The lower adoption of Po in this study may reflect procedural complexity, limited material availability, and the clinical decision-making abilities of undergraduate students, who may prefer Pp due to its predictability and familiarity with procedural steps during urgent care. A recent mixed-methods study found that dental students report lower confidence and higher anxiety in endodontic emergency care, with confidence improving only as case exposure increases—factors that may favor their selection of familiar procedures such as Pp [28].

SAP can itself produce substantial pain, particularly on biting and chewing, and may accompany several pulpal conditions, including SIP, AIP, NEC, PI, or PT cases, as observed in this study [4]. In addition to Pp and Po, OD has been shown to further reduce pain in teeth with SIP and SAP, with improvement reported within the first three days [25]. Some evidence suggests that Po combined with OD can relieve chewing pain earlier than Pp alone [29]. In the present study, a notable proportion of urgent cases were PT with SAP, consistent with prior reports [27]. Failures in PT teeth may arise from coronal leakage, improper seal, missed canals, root fracture, or periodontal issues. With increasing patient preference for retaining natural teeth, demand for non-surgical retreatment continues to grow [30]. PI cases were also noted in this study, similar to findings from the Queensland study, which reported a female predominance [22]. In our cohort, however, PI cases were evenly distributed across genders. Clinically, these presentations likely reflect inter-appointment urgencies or missed follow-ups that later present with persistent or recurrent symptoms. The lower odds of returning among AIP/SAP patients suggest they were less likely to complete the RCT than the overall urgent-care group. This may be attributed to the relatively milder symptoms of SAP, given that AIP is asymptomatic [4]. Additionally, initial urgent treatment may have provided sufficient relief, reducing perceived need for further care.

The present study demonstrated a high follow-up rate among patients who received urgent RCT, with no gender-based differences in return patterns. Although previous investigations have reported that relief of acute symptoms after urgent treatment may lead some patients to discontinue care, this trend was not evident in our cohort [17]. Dental insurance policies also influence follow-up, as patients often attend appointments in accordance with policy requirements [17]. Socioeconomic and insurance-related barriers are known contributors to incomplete care; for example, two US studies reported that 41.5–52.4% of Medicaid adult patients with dental urgencies did not see a dentist within six months of the emergency visit [31,32]. Insurance limitations in which emergency care is covered but definitive procedures, such as RCT, require prior authorization are frequently cited as reasons for treatment abandonment [17]. In contrast, all patients in the present study received free care at the dental college, eliminating financial and insurance restrictions commonly associated with incomplete treatment [33]. This likely contributed to the higher follow-up rates observed and reduced socioeconomic disparities that typically influence completion of endodontic care. These findings should therefore be regarded as specific to our university setting and exploratory in nature. Studies from other institutional settings have shown mixed patterns. At a US military School, incomplete RCT was more common among patients presenting with preoperative pain, whereas at Rutgers School of Dentistry, 66% of patients who received palliative endodontic care did not attend their scheduled follow-up [14,17]. When comparing broader RCT completion rates regardless of emergency, both these schools reported a completion rate of 77% [14,17]. Similarly, a study from Queensland University reported an 86% RCT recall rate; however, prior urgent care did not increase the likelihood of completing the procedure, aligning with our study’s findings that urgent intervention alone does not ensure follow-up [18]. Other structural elements, such as the number of required visits, may also influence follow-up behavior, as multiple appointments were a significant barrier, particularly for molars, in that study.

The large sample size in the present study provides a reliable basis for analyzing patient characteristics and outcomes. However, several limitations should be considered when interpreting the findings. The data were obtained from a single dental teaching institute, which may limit generalizability and applicability to other settings or institutions with different patient demographics, insurance structures, and clinical workflows. The retrospective design and convenience sampling may introduce selection bias, as diagnoses were based on existing records rather than real-time clinical assessment, thereby limiting verification of true endodontic emergencies. Documentation of systemic involvement and urgency severity was inconsistent, making it difficult to distinguish true emergencies from urgent cases. To avoid misclassification, we categorized all cases as urgent rather than emergent, an important consideration when interpreting the findings. This approach likely produced a heterogeneous sample with variable symptom severity. Because similar scenarios are often labeled as emergencies in the literature, our conservative classification limits direct comparison with studies that use stricter definitions and reduces generalizability to settings with standardized criteria for endodontic emergencies. The results should therefore be interpreted within this context. Another limitation is the restricted set of variables available in the retrospective database. Important factors known to influence completion of endodontic treatment, such as age, socioeconomic status, education level, distance from the clinic, and treatment stage at the emergency visit, were not recorded and therefore could not be assessed. Their absence limits the ability to fully explain follow-up behavior and may obscure important confounding effects. Additionally, small sample sizes in certain diagnostic groups (such as PT/AAA and PI/AAA) produced wide CIs, reducing interpretability. Follow-up completion was measured only by return to the same institution and does not account for patients who may have completed treatment elsewhere. Finally, as a teaching institute, treatment decisions may reflect students’ experience levels, material availability, and instructors’ preferences, which could influence treatment modality selection.

Future research should employ prospective designs and consider gathering socioeconomic and geographic data. Cross-sectional studies exploring reasons for missed appointments and barriers to follow-up would also be valuable. Future investigations should evaluate the effectiveness of urgent treatments, such as Po and Pp, in achieving long-term pain relief. Additionally, comparing treatment outcomes between patients treated by undergraduate students and postgraduate students, or between general dentists and endodontists, could provide valuable insights into the influence of experience, training, and educational background on patient outcomes.

## 5. Conclusions

Within the limitations of this study, most urgent endodontic cases in this university setting involved symptomatic disease, primarily SIP and SAP, particularly in posterior teeth. Gender and region did not influence return rates, but mandibular teeth demonstrated higher odds of follow-up. Although the study showed good follow-up rates, a substantial 27.2% of the urgent cases did not return. Two clinical scenarios were associated with a lower likelihood of returning: diagnosis of AIP/SAP and cases managed by CD/OD. However, these patterns should be interpreted as associations rather than predictors, given the free-care, single-center, retrospective design. Further validation through multi-site, prospective studies is needed.

## Figures and Tables

**Table 1:** Diagnostic variables based on AAE classification seen in patients with urgent care.

Category	Clinical Features	Radiographic Features
SIP	Spontaneous pain; lingering thermal sensitivity; may have referred pain	Usually normal early; may progress later
AIP *	No symptoms; deep caries or trauma likely to expose pulp	May show caries close to pulp
NEC *	No response to vitality tests; asymptomatic	May show periapical changes if infected
PT *	Endodontically treated; no response to pulp tests	Obturated canals
PI *	Partial endodontic treatment with the presence of a coronal restoration (temporary/permanent)	Variable (may show intracanal radiopacity if medicament was placed)
NAT ^#^	No pain on percussion/palpation	Intact lamina dura; uniform PDL space
SAP	Pain on biting/percussion; may be severe	May show widened PDL or apical radiolucency
AAP ^#^	Typically, asymptomatic	Apical radiolucency present
AAA	Rapid onset; severe pain and swelling; possible systemic symptoms	Radiographic signs may be absent in early stages, or may show radiolucency of varying dimensions

SIP, Symptomatic Irreversible pulpitis; SAP, Symptomatic Apical Periodontitis; AAP, Asymptomatic Irreversible Pulpitis; NAT, Normal Apical Tissue; PT, Previously Treated; AAA, Acute Apical Abscess; PI, Previously Initiated; NEC, Necrotic Pulp; AIP, Asymptomatic Irreversible Pulpitis; *, pulpal diagnosis seen with associated periapical symptoms; ^#^, periapical diagnosis seen with associated pulpal symptoms.

**Table 2:** Treatment patterns provided for urgent care.

Category	Treatment Description
Pp	The removal of pulpal tissue from the root canal space, either completely or partially, with or without placement of intracanal medicament
Po	Removal of only the coronal portion of the pulp (partial or complete), with or without placement of intracanal medicament
ReTx	Involves removal of the previous root canal filling material and temporization with or without placement of an intracanal medicament
ReTx/OD	Involves removing the previous root canal filling and temporizing, with or without an intracanal medicament, while reducing the coronal tooth structure to prevent occlusion with the opposing tooth.
CD	Cleaning and shaping the canals and temporization with or without intracanal medicament
CD/OD	Cleaning and shaping the canals and temporization with or without an intracanal medicament, with reduction of the coronal tooth structure to keep it out of occlusion with the opposing tooth
I&D	Cleaning and shaping the canals and temporization with or without intracanal medicament, with a small incision at the site of maximum swelling to drain pus, relieve pressure, and reduce pain

Pp, Pulpectomy; Po, Pulpotomy; ReTx, Retreatment; ReTx/OD, Retreatment with Occlusal reduction; CD, Canal debridement; CD/OD, Canal debridement with Occlusal reduction; I&D, Incision and drainage.

**Table 3:** Comparison of urgent and non-urgent endodontic cases by gender, arch, and region.

		Urgent	%	Non-Urgent	%	χ^2^	*p*-Value
Gender	Male	378	46.6%	354	43.9%	1.178	0.278 ns
	Female	434	53.4%	453	56.1%		
Arch	Maxilla	429	52.8%	411	50.9%	0.587	0.443 ns
	Mandible	383	47.2%	396	49.1%		
Region	Anterior	301	37.1%	305	37.8%	0.091	0.763 ns
	Posterior	511	62.9%	502	62.2%		

χ^2^, chi-square test; ns, non-significant difference with *p*-value ≤ 0.05.

**Table 4:** Comparison of gender-based distribution across dental arches and regions.

Variable		Male	Female	χ^2^	*p*-Value
Arch	Maxilla	215	214	4.64	0.031 *
		56.9%	49.3%		
	Mandible	163	220		
		43.1%	50.7%		
Region	Anterior	163	138	11.10	<0.001 **
		43.1%	31.8%		
	Posterior	215	296		
		56.9%	68.2%		

χ^2^, chi-square test; *, statistically significant difference with *p*-value ≤ 0.05; **, highly significant difference with *p*-value ≤ 0.05.

**Table 5:** Association between diagnosis and demographic/anatomical variables.

		SIP/ SAP	SIP/ AAP	SIP/ NAT	PT/ SAP	PT/ AAA	PI/ SAP	PI/ AAA	NEC/ AAA	NEC/ SAP	AIP/ SAP	χ^2^	*p*-Value
Gender	Male	156	69	49	44	1	23	2	4	14	16	7.22	0.614
		45%	51.5%	43.8	43.6%	100%	43.4%	50%	66.7%	50%	61.5%		
	Female	191	65	63	57	0	30	2	2	14	10		
		55%	48.5%	56.3%	56.4%	0	56.6%	50%	33.3%	50%	38.5		
Arch	Maxilla	173	64	58	61	1	29	3	5	17	18	12.48	0.187
		49.9%	47.8%	51.8%	60.4%	100%	54.7%	75.0%	83.3%	60.7%	69.2%		
	Mandible	174	70	54	40	0	24	1	1	11	8		
		50.1%	52.2%	48.2%	39.6%	0.0%	45.3%	25.0%	16.7%	39.3%	30.8%		
Region	Anterior	122	45	43	44	1	13	3	6	11	13	22.99	0.006 *
		35.2%	33.6%	38.4%	43.6%	100%	24.5%	75.0%	100.0%	39.3%	50.0%		
	Posterior	225	89	69	57	0	40	1	0	17	13		
		64.8%	66.4%	61.6%	56.4%	0.0%	75.5%	25.0%	0.0%	60.7%	50.0%		

SIP/SAP, Symptomatic Irreversible pulpitis with Symptomatic Apical Periodontitis; SIP/AAP, Symptomatic irreversible Pulpitis with Asymptomatic Irreversible Pulpitis; SIP/NAT, Symptomatic Irreversible Pulpitis with Normal Apical Tissue; PT/SAP, Previously Treated with Symptomatic Apical Periodontitis; PT/AAA, Previously Treated with Acute Apical Abscess; PI/SAP, Previously Initiated with Symptomatic Apical Periodontitis; PI/AAA, Previously Initiated with Acute Apical Abscess; NEC/SAP, Necrotic pulp with symptomatic apical periodontitis; NEC/AAA, Necrotic pulp with Acute Apical Abscess; AIP/SAP, Asymptomatic Irreversible Pulpitis with Symptomatic Apical Periodontitis; *, statistically significant difference with *p* value ≤ 0.05.

**Table 6:** Comparison of urgent treatment modalities by Gender, Arch and Region with χ^2^ tests.

		Pp	Po	ReTx	ReTx/OD	I&D	CD/OD	CD	χ^2^	*p*-Value
Gender	Male	226	48	18	26	7	22	31	2.52	0.866
		46%	47.06%	40%	46.43%	64%	50.00%	49%		
	Female	265	54	27	30	4	22	32		
		54%	52.94%	60.00%	53.57%	36.36%	50.00%	51%		
Arch	Maxilla	260	36	38	23	9	23	40	40.33	<0.001 *
		52.96%	35.29%	84.40%	41.07%	82%	52.27%	63.49%		
	Mandible	231	66	7	33	2	21	23		
		47.04%	64.71%	15.60%	58.93%	18.18%	47.73%	36.51%		
Region	Anterior	188	38	40	4	10	3	50	142.18	<0.001 *
		38.29%	37.26%	88.90%	7.14%	91%	6.82%	79.37%		
	Posterior	303	64	5	52	1	41	13		
		61.71%	62.74%	11.10%	92.86%	9.09%	93.18%	20.63%		

Pp, Pulpectomy; Po, Pulpotomy; ReTx, Retreatment; ReTx/OD, Retreatment with Occlusal Reduction; I&D, Incision & Drainage; CD/OD, Canal Debridement with Occlusal Reduction; CD, Canal Debridement; χ^2^, Pearson chi-square test; *, Statistically significant difference with *p* ≤ 0.05.

**Table 7:** Multivariable ridge-penalized logistic regression of follow-up: Effects of gender, arch, region, and diagnosis.

Predictor	Level (vs. Reference)	aOR	95% CI	*p*-Value
Gender	Female (vs. Male)	1.07	0.82–1.48	0.79
Arch	Mandible (vs. Maxilla)	1.57	1.16–2.07	<0.01 *
Region	Posterior (vs. Anterior)	0.95	0.73–1.20	0.54
Diagnosis	SIP/AAP (vs. SIP/SAP)	0.99	0.66–1.39	0.53
	SIP/NAT	1.05	0.76–1.67	0.81
	PT/SAP	0.82	0.52–1.12	0.17
	PT/AAA	1.00	1.00–1.08	0.09
	PI/SAP	0.82	0.47–1.24	0.15
	PI/AAA	1.00	0.34–1.71	0.29
	NEC/AAA	1.00	0.71–2.96	0.75
	NEC/SAP	0.77	0.37–1.28	0.17
	AIP/SAP	0.61	0.24–1.00	0.04 *

aOR, adjusted Odds Ratio; CI, Confidence Interval; two-sided *p*-values using nonparametric bootstrap resampling (B = 200); *, Statistical significance with *p* ≤ 0.05. Reference categories: Male, Maxilla, Anterior, SIP/SAP.

**Table 8:** Multivariable ridge-penalized logistic regression of follow-up: Effects of gender, arch, region, and treatment.

Predictor	Level (vs. Reference)	aOR	95% CI	*p*-Value
Gender	Female (vs. Male)	1.08	0.79–1.49	0.620
Arch	Mandible (vs. Maxilla)	1.68	1.21–2.34	0.0019 *
Region	Posterior (vs. Anterior)	1.04	0.72–1.49	0.840
Treatment	Po (vs. Pp)	0.79	0.48–1.28	0.334
	ReTx	0.64	0.32–1.25	0.188
	ReTx/OD	0.83	0.44–1.57	0.568
	CD	1.12	0.60–2.09	0.728
	CD/OD	0.29	0.15–0.55	<0.001 *

aOR, adjusted Odds Ratios from non-penalized maximum-likelihood logistic regression; 95% CIs, confidence intervals from Wald intervals on the log-odds scale, exponentiated to the OR scale; *p*-values for Wald tests; *, Statistical significance with *p* ≤ 0.05. Reference categories: Male, Maxilla, Anterior, PP.

**Table 9:** Follow-up rates after urgent endodontic care: odds analysis along with logistic regression analysis to find the odds ratios across diagnostic categories.

Diagnosis	n	Follow-Up	Non-Follow-Up	Odds (FU/Non-FU) (95% CI)	OR vs. Overall (95% CI)	*p* (Wald)
Overall	812	591	221	2.67 (2.29–3.12)	Reference	-
SIP/SAP	347	261	86	3.03 (2.38–3.87)	1.13 (0.85–1.51)	0.179
		44.20%	38.90%			
SIP/AAP	134	99	35	2.83 (1.92–4.16)	1.06 (0.69–1.60)	0.755
		16.80%	15.80%			
SIP/NAT	112	84	28	3.00 (1.96–4.60)	1.12 (0.71–1.77)	0.557
		14.20%	12.70%			
PT/SAP	101	69	32	2.16 (1.42–3.28)	0.81 (0.52–1.26)	0.282
		11.70%	14.50%			
PI/SAP	53	39	14	2.79 (1.51–5.13)	1.04 (0.55–1.96)	0.892
		6.60%	6.30%			
NEC/SAP	28	16	12	1.33 (0.63–2.82)	0.50 (0.23–1.07)	0.063
		2.70%	5.40%			
AIP/SAP	26	14	12	1.17 (0.54–2.52)	0.44 (0.20–0.96)	0.032 *
		2.40%	5.40%			
PI/AAA	4	3	1	3.00 (0.31–28.84)	1.12 (0.12–10.84)	0.921
		0.50%	0.50%			
NEC/AAA	6	5	1	5.00 (0.58–42.80)	1.87 (0.22–16.09)	0.566
		0.80%	0.50%			
PT/AAA ^#^	1	1	0	3.00 (0.12–73.65)	1.12 (0.05–27.64)	0.942
		0.20%	0			

SIP/SAP, Symptomatic Irreversible pulpitis with Symptomatic Apical Periodontitis; SIP/AAP, Symptomatic irreversible Pulpitis with Asymptomatic Irreversible Pulpitis; SIP/NAT, Symptomatic Irreversible Pulpitis with Normal Apical Tissue; PT/SAP, Previously Treated with Symptomatic Apical Periodontitis; PT/AAA, Previously Treated with Acute Apical Abscess; ^#^, A Haldane–Anscombe correction (+0.5 to each cell) was applied due to a zero cell; PI/SAP, Previously Initiated with Symptomatic Apical Periodontitis; NEC/SAP, Necrotic Pulp with Symptomatic Apical Periodontitis; AIP/SAP, Asymptomatic Irreversible Pulpitis with Symptomatic Apical Periodontitis; OR, Odds ratio; FU, Follow-up; CI, Confidence Interval with the level of confidence at 95%; *p*-Walds method with <0.05 as significant; *, statistically significant difference with *p*-value ≤ 0.05.

**Table 10:** Follow-up After Urgent Care by Treatment Modality: Odds and Logistic Regression (Wald).

Diagnosis	n	Follow-Up	Non-Follow-Up	Odds Analysis (95% CI)	OR (95% CI)	*p* (Wald)
Overall	812	591	221	2.67	Reference	*-*
				(2.29–3.12)		
Pp	491	370	121	3.06	1.14	0.042 *
	60.47%	62.61%	54.75%	2.49–3.75	0.93–1.40	
Po	102	74	28	2.64	0.99	0.955
	12.56%	12.52%	12.67%	1.72–4.07	0.64–1.52	
ReTx	45	28	17	1.65	0.62	0.105
	5.54%	4.74%	7.69%	0.91–2.98	0.34–1.12	
ReTx/OD	56	41	15	2.73	1.02	0.944
	6.90%	6.94%	6.78%	1.53–4.90	0.57–1.83	
I&D	11	9	2	4.5	1.68	0.503
	1.35%	1.52%	0.90%	1.10–18.47	0.41–6.91	
CD/OD	44	21	23	0.91	0.34	<0.001 *
	5.41%	3.55%	10.41%	0.51–1.64	0.19–0.61	
CD	63	48	15	3.2	1.2	0.527
	7.76%	8.12%	6.78%	1.81–5.67	0.68–2.12	

Pp, Pulpectomy; Po, Pulpotomy; ReTx, Retreatment; ReTx/OD, Retreatment with Occlusal Reduction; I&D, Incision & Drainage; CD/OD, Canal Debridement with Occlusal Reduction; CD, Canal Debridement; CI, Confidence interval; *, Statistically significant difference with *p* ≤ 0.05.

## Data Availability

The original contributions presented in this study are included in the article. Further inquiries can be directed to the corresponding author.

## Abbreviations

The following abbreviations are used in this manuscript:

AAA	Acute Apical Abscess
AAP	Asymptomatic Apical Periodontitis
AIP	Asymptomatic Irreversible Pulpitis
CI	Confidence Interval
CD	Canal Debridement
I&D	Incision and Drainage
NAT	Normal Apical Tissue
OD	Occlusal Reduction
OR	Odds Ratio
PI	Previously Initiated
NEC	Pulp Necrosis
Po	Pulpotomy
Pp	Pulpectomy
PT	Previously Treated
RCT	Root Canal Treatment
ReTx	Retreatment
SAP	Symptomatic Apical Periodontitis
SIP	Symptomatic Irreversible Pulpitis
